# Temporal Trend of Carpal Tunnel Release Surgery: A Population-Based Time Series Analysis

**DOI:** 10.1371/journal.pone.0097499

**Published:** 2014-05-14

**Authors:** Naif Fnais, Tara Gomes, James Mahoney, Sami Alissa, Muhammad Mamdani

**Affiliations:** 1 College of Medicine, King Saud University, Riyadh, Saudi Arabia; 2 The Institute for Clinical Evaluative Sciences, Toronto, Ontario, Canada; 3 Leslie Dan Faculty of Pharmacy, University of Toronto, Toronto, Ontario, Canada; 4 Division of Plastic & Reconstructive Surgery, Saint Michael’s Hospital, Toronto, Ontario, Canada; 5 Division of Plastic & Reconstructive Surgery, University of Toronto, Toronto, Ontario, Canada; 6 Applied Health Research Centre, St. Michael’s Hospital, Toronto, Ontario, Canada; 7 The Keenan Research Centre of the Li KaShing Knowledge Institute of St. Michael’s Hospital, Toronto, Ontario, Canada; 8 Department of Health Policy, Management, and Evaluation, University of Toronto, Toronto, Ontario, Canada; University of Sheffield, United Kingdom

## Abstract

**Background:**

Carpal tunnel release (CTR) is among the most common hand surgeries, although little is known about its pattern. In this study, we aimed to investigate temporal trends, age and gender variation and current practice patterns in CTR surgeries.

**Methods:**

We conducted a population-based time series analysis among over 13 million residents of Ontario, who underwent operative management for carpal tunnel syndrome (CTS) from April 1, 1992 to March 31, 2010 using administrative claims data.

**Results:**

The primary analysis revealed a fairly stable procedure rate of approximately 10 patients per 10,000 population per year receiving CTRs without any significant, consistent temporal trend (p = 0.94). Secondary analyses revealed different trends in procedure rates according to age. The annual procedure rate among those age >75 years increased from 22 per 10,000 population at the beginning of the study period to over 26 patients per 10,000 population (p<0.01) by the end of the study period. CTR surgical procedures were approximately two-fold more common among females relative to males (64.9% vs. 35.1 respectively; p<0.01). Lastly, CTR procedures are increasingly being conducted in the outpatient setting while procedures in the inpatient setting have been declining steadily – the proportion of procedures performed in the outpatient setting increased from 13% to over 30% by 2010 (p<0.01).

**Conclusion:**

Overall, CTR surgical-procedures are conducted at a rate of approximately 10 patients per 10,000 population annually with significant variation with respect to age and gender. CTR surgical procedures in ambulatory-care facilities may soon outpace procedure rates in the in-hospital setting.

## Introduction

Carpal tunnel syndrome (CTS) is an entrapment neuropathy, which is caused mainly by median nerve compression and irritation at the level of carpal tunnel [1]. Symptoms of CTS include pain and paraesthesia in the wrist and hand that can radiate to the forearm [2], [3]. CTS is said to affect 1% to 3% of population [4], [5], with higher incidence in certain occupational groups who perform repetitive motions of the hand and wrist such as automobile assembly workers [6], and those with medical conditions such as renal failure and diabetes mellitus [7]–[9].

Previous study suggests the prevalence of CTS in the general female population aged 25 to 74 years to be approximately 9%, in comparison to a much lower prevalence of 0.6% among men from the same age group [10]. In Sweden, Atroshi et al, suggest that the prevalence of CTS in the general population is 3.8% for clinically diagnosed cases and 2.7% for electrophysiologically confirmed cases [5]. A more recent American study showed that the overall age and sex adjusted incidence of CTS is 376 per 100,000 person-years (95% confidence interval [CI], 369–384), with much greater incidence in women (491 per 100,000 person-years; 95% CI, 479–502) than men (258 per 100,000 person-years; 95% CI, 249–268) [11].

Treatment of CTS involves a variety of interventions including non-surgical and surgical options. The decision is made according to the severity of the symptoms. Patients with mild to moderate CTS can be offered non-surgical treatment, which includes splinting, exercises, corticosteroid injection, oral medications and vitamins. Surgical treatment is offered to those who have severe and persistent CTS, which might be associated with functional and occupational disturbance. Surgical treatment includes open or endoscopic release of the carpal tunnel.

Carpal tunnel release (CTR) surgical procedure is the most common hand and wrist surgery in the USA, with over 400,000 procedures per year [12]–[14] and relatively high social and economic costs that exceed USD 2 billion, annually [11], [14]. In Italy, the annual incidence of CTR was reported to be 13.9 per 10,000 person-years for men and 50.6 per 10,000 person-years for women [15]. In 1988, a study from Ontario, Canada, showed higher rates in the general population with 37 per 10,000 women aged 50 to 55 years [16].

As with many surgical procedures, the cost CTR surgical procedure varies depending on the setting [in-hospital vs. out-of-hospital (ambulatory care)] and type of procedure. The average cost of in-hospital procedure is USD 5,480 vs. USD 2,491 for the out-of-hospital procedure [17]. Moreover, the use of the main operating room for CTR is almost four times as expensive, and less than half as efficient as CTR in an ambulatory setting [18], [19]. The higher costs of in hospital procedures can be explained by the longer duration of surgery, the need for more operating room personal, the presence of anesthesiologists, the type anesthesia (wide awake approach v.s. full sedation), and the expense of surgical supplies [18].

Although CTS has been widely investigated, little is known about the pattern of CTR surgical procedures. In this study, we aimed to investigate temporal trends of CTR surgical procedures as well as the impact of age, gender and practice setting on these rates.

## Materials and Methods

### Study Design and Setting

We conducted a population-based cross-sectional time series analysis among over 13 million Ontario residents using healthcare administrative databases, to examine the incidence of operative management of CTS between April 1, 1992 and March 31, 2010. All subjects had universal access to healthcare services such as hospital care and physician services. This study received approval from the Research Ethics Board of Sunnybrook Health Sciences Centre, Toronto, Canada.

### Data Sources

We used the Canadian Institute for Health Information Discharge Abstract Database to define all patients undergoing operative management of CTS over the study period. This database contains detailed diagnostic and procedural information for all inpatient hospital admissions and same-day surgeries in Ontario. We used the Ontario Health Insurance Plan (OHIP) database to identify claims for inpatient and outpatient physician services. The Institute for Clinical Evaluative Sciences is a prescribed entity under Ontario’s Personal Health Information Protection Act (PHIPA) and is allowed to house Ontario’s administrative claims databases used in this study for research purposes without patient consent. The ethics review board acknowledges this status and does not require patient consent for these studies. Ontario population estimates for each year were obtained from Statistics Canada (Statistics Canada. Available at: http://www.statcan.ca). Basic demographic information was obtained from the Registered Persons Database, which contains a unique entry for each Ontario resident who has ever received a health card. The databases were linked in an anonymous fashion using encrypted 10-digit health-card numbers, and are routinely used for population-based healthcare research [20], [21].

### Identification of Patients and Procedure Rates

We identified all Ontario residents aged between 15 and 95 years who underwent operative management of CTS over the study period using the OHIP fee code N290. NCS confirming CTS was identified using the OHIP fee code G466 [22]. We excluded individuals with missing age and gender and those aged younger than 15 years or older than 95 years at the time of surgery from our cohort of patients.

### Statistical Analysis

Time-series analysis was used to examine annual patterns in CTR surgical procedure rates over the study period. Exponential smoothing models and autoregressive integrated moving average (ARIMA) models were used to assess temporal trends over time. To assess model appropriateness in our analysis, we used the autocorrelation functions and the augmented Dickey–Fuller test [23]. Autocorrelation, partial autocorrelation and inverse autocorrelation were assessed for model-parameter appropriateness and seasonality. The presence of “white noise” was assessed by examining the autocorrelations at various lags, using the Ljung–Box χ^2^ statistic. All p values were considered significant at a level 0.05.

Patient age at the time of surgery was divided into four age groups (15–35, 36–55, 56–75, 76–95). Age-specific rates overall, and stratified by women and men were calculated for each procedure using the Ontario population for the relevant year as the denominator. Out-of-hospital procedures included any CTR surgical procedure performed outside the operating room, including office-based procedures. Procedure rates for 1992 and 2010 are not reported, as data from the first quarter of 1992 and second to fourth quarters of 2010 were not available at the time of study.

All data were compiled and analysed using SAS version 9.2 (2008; SAS Institute Inc., Cary, North Carolina, USA).

## Results

Over the 18 year study period, 253,240 carpal tunnel release surgeries were performed in Ontario among a population exceeding 13 million adults. Of these, 205,771 (81.3%) were performed as in-hospital procedures and 47,469 (18.7%) were performed out of the hospital setting. The majority of procedures (N = 164,371; 64.9%) were conducted among female patients, and approximately 13% (33,711) of patients undergoing CTR surgical procedure had a NCS prior to their procedure. Baseline characteristics are shown in Table 1.

**Table 1:** Characteristics of Carpal Tunnel Surgery Patients: All Surgeries between Jan 1 1993 and December 31 2009.

	N	%
**Total Number of Surgeries**	253,240	
**Age: Mean (SD)**	54.66 (15.56)	
**Age Category**		
* 15 to 35*	26,019	10.27
* 36 to 55*	116,209	45.89
* 56 to 75*	79,622	31.44
* 76 to 95*	31,390	12.40
**Male Gender**	88,869	35.09
**Location of Surgery**		
* In Hospital*	205,771	81.26
* Out of Hospital*	47,469	18.74
**Prior NCS**		
* Yes*	33,711	13.31
* No*	219,529	86.69
**Number of CTS OHIP claims on date of surgery**		
* 1*	142,863	56.41
* 2*	82,937	32.75
* 3*	27,414	10.83
* 4*	26	0.01

**Abbreviations:** N: Number; SD: Standard Deviation; NCS: Nerve Conduction Study; CTS: Carpal Tunnel Syndrome; OHIP: Ontario Health Insurance Plan.

### Trends in Surgery Procedure Rates over Time

The primary analysis revealed a fairly stable annual CTR surgical procedure rate of approximately 10 patients per 10,000 population without any significant temporal trend (p = 0.94; table 1).

### Surgery Rates and Age

**Figure 1:**
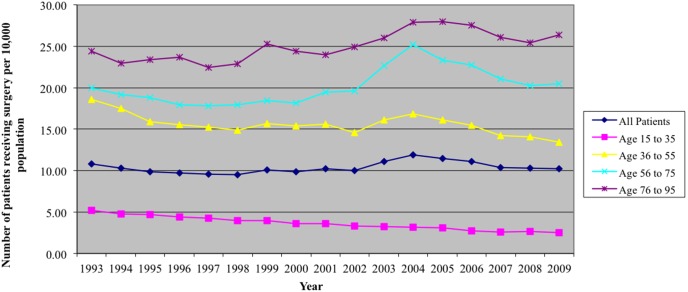
Patient Surgery Rates, stratified by age (per 10,000 population).

In a secondary analysis stratified by age, there was a significantly higher procedure rate among older patients relative to younger patients (p<0.01). Procedure rates for the age group older than 75 years increased to over 25 patients per 10,000 population per year (p<0.01), whereas the rate for those aged 15–35 years declined over time to approximately 3 per 10,000 population per year (p<0.01). Despite being the highest risk group for CTS, there was a decline in the annual procedure rates among patients aged 36 to 55 years from 18.6 to nearly 13.4 per 10,000 population (p<0.01; figure 1) over the study period.

### Surgery Rates and Gender

**Figure 2:**
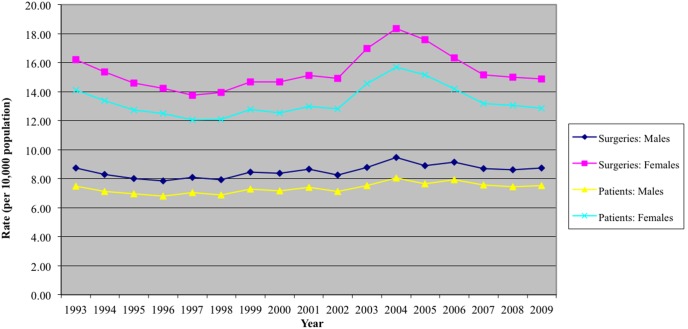
Surgery and Patient Rates, stratified by gender (per 10,000 population).

On average, CTR surgical procedures were approximately two-fold more common among females relative to males (16.0 vs. 8.7 per 10,000 population per year respectively; p<0.01; figure 2).

### Surgery Rates and Hospital Location

**Figure 3:**
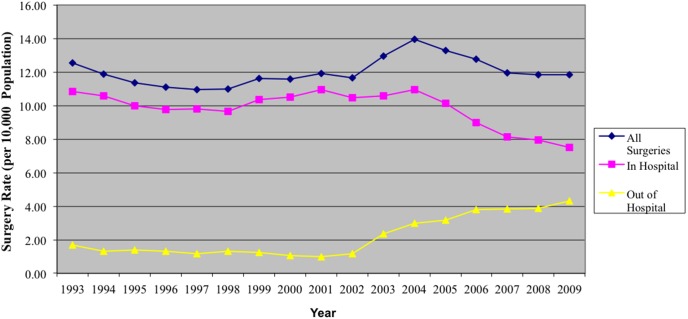
Surgery Rates, Stratified by Location (in vs. out-of-hospital).

The annual rate of ambulatory CTR surgical procedures increased from 1.7 per 10,000 population in 1993 to 4.3 per 10,000 population in 2009, with a dramatic rise beginning in 2002 (p<0.01). Conversely, in-hospital procedure decreased significantly over time after 2004 from 11 to 7.5 per 10,000 population in 2009 (p<0.01; figure 3).

## Discussion

The findings of our study spanning eighteen-years suggest a consistent rate of CTR surgical procedures over time in Ontario. Higher rates of CTR were noted in females and older patients and the majority of CTR surgical procedures were performed without a prior NCS. To our knowledge, this is the largest study examining trends in carpal tunnel release conducted.

Our study demonstrated that ambulatory CTR surgical procedure has recently increased in prevalence and may become a more common occurrence than in-hospital surgery. Indeed, Farajado et al, reported a 38% increase in ambulatory CTR surgical procedures between 1996 and 2006 in the USA, where ambulatory procedures are now predominant [24]. CTR in the ambulatory care setting is generally less expensive and more efficient than in-hospital surgeries [18], [19], however the persistence of higher in-hospital procedure rates may be attributable to the lack of clear regulations and guidelines on the management of CTS. Such regulations could determine the surgical technique and setting for CTR surgical procedure [24]. The shift in surgery location demonstrated in our study may have a substantial economic impact as this change could reduce the impact of CTR surgical procedure on hospital resources and health care costs.

In contrast to previous reports, an interesting finding in the present study was that the highest CTR surgical procedure rate standardized for population growth was among patients older than 75 years [24]–[27]. This may be due to higher comorbid conditions that older patients tend to have in comparison to younger age groups or progression of previously undiagnosed CTS at an earlier age.

**Figure 4:**
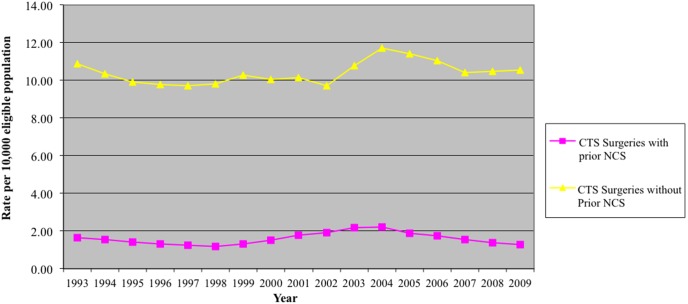
NCS and surgeries rate.

NCS remains the diagnostic test of choice in the diagnosis of CTS. When a patient presents with symptoms mimicking CTS, NCS can help to rule out other causes. However, 16–34% of clinically defined CTS can be missed with NCS [28]. According to the American Academy of Orthopedic Surgeons (AAOS) clinical practice guidelines on the diagnosis of carpal tunnel syndrome, “ *The physician should obtain electrodiagnostic tests if clinical and/or provocative tests are positive and surgical management is being considered* ” [29]. Although our study timeframe was largely limited to prior to the AAOS recommendations, the fact that only 13% of the CTR cases in our study had NCS prior to surgery is unexpected, which indicates that surgeons in Ontario rely mainly on their clinical judgment in the diagnosis and management of CTS patients (figure 4). This is consistent with previous reports that suggest the diagnosis of CTS may not change with the use of NCS [30].

Some limitations of our study merit emphasis. The accuracy of coding for CTR surgical procedure has not been validated in our databases and therefore we may not have identified all surgeries conducted during the study period. However, it is unlikely that the coding validity would differ between in and out-of-hospital surgeries and this would only lead to underestimation of our reported surgery rates. We also lacked detailed information on the reasons driving the choice of setting for CTR surgical procedure (i.e. inpatient vs out-of-hospital) to better understand the observed trends. Furthermore, because this data is collected for physician billing purposes, it is likely complete and of high quality.

CTR surgical procedures are conducted at a rate of approximately 10 patients per 10,000 population annually with significant variation with respect to age and gender. The increasing trend to perform CTR surgical procedures in ambulatory care facilities may soon outpace procedure rates in the inpatient hospital setting.
